# LOX-1, OxLDL, and Atherosclerosis

**DOI:** 10.1155/2013/152786

**Published:** 2013-07-10

**Authors:** Angela Pirillo, Giuseppe Danilo Norata, Alberico Luigi Catapano

**Affiliations:** ^1^Center for the Study of Atherosclerosis, E. Bassini Hospital, 20092 Cinisello Balsamo, Italy; ^2^IRCCS Multimedica, 20162 Milan, Italy; ^3^Department of Pharmacological and Biomolecular Sciences, Università degli Studi di Milano, 20133 Milan, Italy; ^4^Centre for Diabetes, The Blizard Institute, Barts and The London School of Medicine & Dentistry, Queen Mary University, London E1 2AT, UK

## Abstract

Oxidized low-density lipoprotein (OxLDL) contributes to the atherosclerotic plaque formation and progression by several mechanisms, including the induction of endothelial cell activation and dysfunction, macrophage foam cell formation, and smooth muscle cell migration and proliferation. Vascular wall cells express on their surface several scavenger receptors that mediate the cellular effects of OxLDL. The lectin-like oxidized low-density lipoprotein receptor-1 (LOX-1) is the main OxLDL receptor of endothelial cells, and it is expressed also in macrophages and smooth muscle cells. LOX-1 is almost undetectable under physiological conditions, but it is upregulated following the exposure to several proinflammatory and proatherogenic stimuli and can be detected in animal and human atherosclerotic lesions. The key contribution of LOX-1 to the atherogenic process has been confirmed in animal models; LOX-1 knockout mice exhibit reduced intima thickness and inflammation and increased expression of protective factors; on the contrary, LOX-1 overexpressing mice present an accelerated atherosclerotic lesion formation which is associated with increased inflammation. In humans, LOX-1 gene polymorphisms were associated with increased susceptibility to myocardial infarction. Inhibition of the LOX-1 receptor with chemicals or antisense nucleotides is currently being investigated and represents an emerging approach for controlling OxLDL-LOX-1 mediated proatherogenic effects.

## 1. Introduction

Atherosclerosis is a chronic inflammatory vascular disease, having as ultimate outcome the atheromatous plaque, a focal lesion located within the intima of large and medium sized arteries [1, 2]. Subendothelial retention of low density lipoprotein (LDL) and its oxidative modification represent the initial event in atherogenesis, which is followed by infiltration and activation of blood inflammatory cells. Oxidized LDL (OxLDL) in fact activates endothelial cells (ECs) by inducing the expression of several cell surface adhesion molecules which mediate the rolling and adhesion of blood leukocytes (monocytes and T cells); after adhesion to the endothelium, leukocytes migrate into the intima in response to chemokines. Monocytes then differentiate into macrophages that upregulate both toll-like receptors (TLRs), involved in macrophage activation, and scavenger receptors (SRs), that internalize apoptotic cell fragments, bacterial endotoxins, and OxLDL, leading to lipid accumulation and foam cell formation [2]. Macrophage activation leads to the release of proinflammatory cytokines, reactive oxygen species (ROS), proteolytic enzymes involved in matrix degradation and thus in atherosclerotic plaque destabilization. T cells respond to local peptide antigens present on the surface of antigen-presenting cells, become activated, and release proinflammatory cytokines [2].

**Figure 1:**
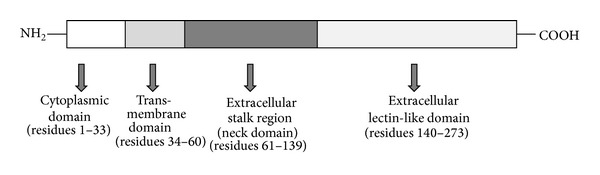
Schematic representation of LOX-1 structure. LOX-1 consists of four domains: a cytoplasmic N-terminal domain, a single transmembrane domain, an extracellular neck domain, and an extracellular lectin-like domain.

**Figure 2:**
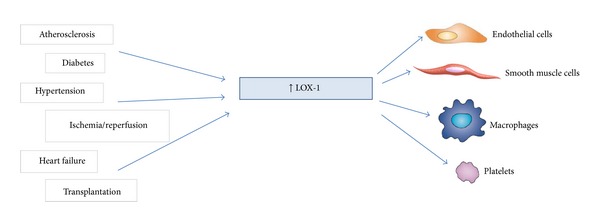
*In vivo* stimuli of LOX-1. Several pathological conditions can upregulate LOX-1 expression resulting in vascular wall cell activation.

OxLDL acts via binding to several SRs, including SR-A, SR-BI, CD36, and lectin-like oxidized low-density lipoprotein receptor-1 (LOX-1) [3]. LOX-1 is a type II integral membrane glycoprotein consisting of a short N-terminal cytoplasmic domain, a transmembrane domain, a neck region, which regulates receptor oligomerization, and an extracellular C-type lectin-like extracellular domain, involved in ligand binding (Figure 1) [4]. LOX-1 has been identified first in ECs as the major OxLDL receptor [5]; however also macrophages and smooth muscle cells express LOX-1 together with other SRs [6]. Basal cellular LOX-1 expression is very low, but it can be induced by several proinflammatory and proatherogenic stimuli [7, 8]. *In vitro*, LOX-1 expression is induced by many stimuli related to atherosclerosis, including proinflammatory cytokines such as tumor necrosis factor alpha (TNF*α*), interleukin-1 (IL-1), and interferon-gamma (IFN*γ*); angiotensin II; endothelin-1; OxLDL and other modified lipoproteins; free radicals; and fluid shear stress [8–10] (Table 1). *In vivo*, the expression of LOX-1 is upregulated in the presence of pathological conditions including atherosclerosis, hypertension, and diabetes [8] (Figure 2). In human atherosclerotic lesions, LOX-1 is overexpressed in ECs especially in the early stage of atherogenesis; in advanced atherosclerotic plaques, LOX-1 is overexpressed in ECs of neovascular formations [11]. Furthermore, LOX-1 is highly expressed by intimal smooth muscle cells (SMCs) and macrophages in human carotid atherosclerotic plaques [11], suggesting the roles for LOX-1 in endothelial activation and in foam cell formation. Finally, the results obtained in LOX-1 knockout or LOX-1 overexpressing mice have suggested a key contribution of LOX-1 in the inflammatory response and lipid deposition in heart vessels [12, 13].

Besides OxLDL, LOX-1 binds multiple ligands, including other forms of modified lipoproteins, advanced glycation end-products, activated platelets, and apoptotic cells [8–10] (Table 2). LOX-1 also binds delipidated OxLDL, suggesting that LOX-1 recognizes the modified apolipoprotein B; furthermore, LOX-1 binds with higher affinity to mildly oxidized LDL rather than extensively oxidized LDL, suggesting the ability to recognize also oxidized lipids (i.e., lipids that are covalently bound to the protein and are not removed by the delipidation process) [3] (Table 2). OxLDL is rapidly internalized into cells following the interaction with LOX-1; this internalization process is inhibited by LOX-1-blocking antibody which acts by preventing the binding of OxLDL with the receptor [14]. After the endocytosis of the complex OxLDL-LOX-1, the receptor is uncoupled from OxLDL, and both are located in separate compartments within the cytosol [14].

LOX-1 is mainly localized in caveolae/lipid rafts (cholesterol-enriched membrane microdomains) in the plasma membrane; its function is modulated by the cholesterol content of membrane: cholesterol depletion induces the mislocalization of LOX-1, which results in a more diffuse distribution of the receptor in the plasma membrane (without a reduction of the amount of receptor exposed on the cell surface) and in a marked reduction of LOX-1-mediated OxLDL binding and uptake [15]. This finding suggests that the clustered distribution of LOX-1 in specific membrane microdomains is essential for an efficient interaction with OxLDL and for the internalization of OxLDL-LOX-1 complexes.

## 2. LOX-1-Mediated Endothelial Dysfunction

### 2.1. LOX-1 Upregulation by OxLDL

**Figure 3:**
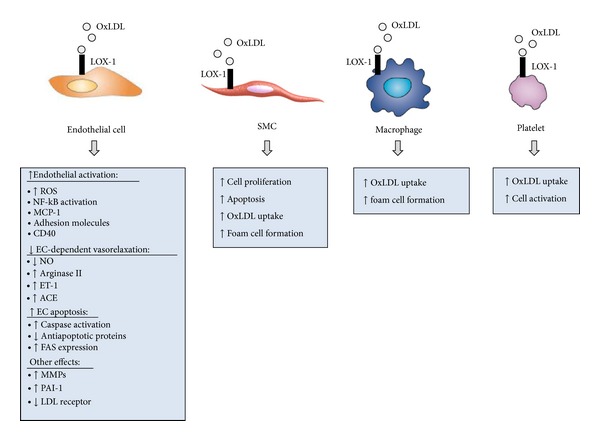
Role of LOX-1 in atherosclerosis. OxLDL binding to LOX-1 induces endothelial activation and dysfunction, supports the recruitment of circulating leukocytes, triggers foam cell formation, and sustains migration and proliferation of smooth muscle cells, thus contributing to the development of the atherosclerotic plaque. Furthermore, OxLDL-LOX-1 interaction may also contribute to plaque destabilization by inducing smooth muscle cell apoptosis and the release of matrix degrading enzymes (MMPs).

Endothelial dysfunction represents a very early stage in the atherogenic process and is a pathological condition characterized by alterations in anti-inflammatory and anticoagulant properties and by impaired ability to regulate vascular tone. LOX-1 is the main receptor for OxLDL in ECs [5], and OxLDL, through LOX-1, contributes to the induction of endothelial dysfunction by several mechanisms (Figure 3). Basal LOX-1 expression is very low, but it can be induced by proinflammatory cytokines generated in a local environment within the arterial wall or by other atherogenic stimuli including OxLDL [8].

OxLDL upregulates LOX-1 mRNA and protein expression in a dose-dependent manner [16]; OxLDL-mediated upregulation of LOX-1 is suppressed by pretreatment of cells with antisense to LOX-1 mRNA [17], suggesting that OxLDL modulates its own receptor through interaction with LOX-1. *In vitro*, LOX-1 expression can be upregulated in ECs also by 15-lipoxygenase-modified LDL, and LOX-1 overexpression increases the association of 15-lipoxygenase-modified LDL to ECs [9], supporting the hypothesis that also minimally oxidized LDL may contribute to LOX-1 induction and to EC activation. LOX-1 upregulation occurs also in ECs exposed to 15-lipoxygenase-modified HDL_3_; this modified lipoprotein is also a ligand for LOX-1 [10].

### 2.2. LOX-1 Mediates OxLDL-Induced EC Activation

After adhesion to the endothelium, monocytes migrate into the intima where they differentiate into macrophages, accumulate lipids, and become foam cells. Recruitment of monocytes involves both chemokines and adhesion molecules. Monocyte chemoattractant protein-1 (MCP-1) is a chemotactic protein for monocytes; incubation of ECs with OxLDL significantly increases MCP-1 expression and monocyte adhesion to ECs; these effects are both suppressed in the presence of an antisense to LOX-1 mRNA [18]. Activation of mitogen-activated protein kinase (MAPK) is required for OxLDL-mediated induction of MCP-1, and antisense to LOX-1 mRNA completely inhibits the OxLDL-induced MAPK activation [18]. Additional chemokines are upregulated by LOX-1 activation in response to OxLDL, including IL-8, chemokine (C-X-C motif) ligands 2 and 3 (CXCL2 and CXCL3) [19].

Upregulation of endothelial adhesion molecules contributes to the leukocyte adhesion; OxLDL significantly increases the expression of E-selectin, P-selectin, vascular cell adhesion molecule-1 (VCAM-1), and intercellular adhesion molecule-1 (ICAM-1) in ECs [20]. These effects are mediated by LOX-1, since antisense to LOX-1 mRNA decreases both LOX-1 expression and adhesion molecule upregulation in response to OxLDL [20]. In addition, pretreatment of ECs with a statin or with polyinosinic acid or carrageenan (two known ligands of LOX-1) lowers OxLDL-induced expression of LOX-1 as well as adhesion molecules, confirming that LOX-1 activation plays an important role in OxLDL-induced expression of adhesion molecules [20, 21]. ICAM-1 expression is upregulated in LOX-1-overexpressing ECs exposed to 15-lipoxygenase-modified LDL [9], providing a role for minimally modified LDL (mmLDL) as proinflammatory particle.

Nuclear factor kappa B (NF-*κ*B), which is activated by several proinflammatory cytokines, modulates the expression of proinflammatory genes, including adhesion molecules, cytokines, and chemokines, and is also involved in LOX-1 upregulation [8, 22]. OxLDL, following the binding with LOX-1, activates NF-*κ*B; preincubation of ECs with anti-LOX-1 antibody markedly attenuates the transcription factor activation [23].

The stimulation of CD40/CD40L signaling results in the induction of proatherogenic pathways (including the expression of adhesion molecules and proinflammatory cytokines) and in EC activation [24]. Incubation of human coronary artery endothelial cells (HCAECs) with OxLDL increases the expression of CD40 and CD40L, while the inhibition of LOX-1 with a blocking antibody reduces the OxLDL-mediated increase of CD40 [25].

### 2.3. OxLDL-LOX-1 Interaction Impairs Endothelium-Dependent Relaxation

ECs, due to their location at the interface between blood and the vessel wall, play a crucial role in the control of vascular tone and homeostasis, through the production of several vasoactive mediators. Among them, nitric oxide (NO) is produced by endothelial NO synthase (eNOS) in response to several stimuli; functions mainly as a vasodilator; inhibits leukocyte-endothelial cell adhesion, platelet adhesion and aggregation, and smooth muscle cell proliferation; and stimulates angiogenesis [26]. In disease conditions, ECs exhibit a reduced NO bioavailability, due to an increased degradation of NO in response to an enhanced oxidative stress [26]. The release of NO by ECs can be reduced by OxLDL: OxLDL displaces eNOS from its caveolae membrane localization by depleting caveolae of cholesterol, thus inhibiting NO generation [27]. Furthermore, OxLDL inactivates NO through an oxidative mechanism by increasing cellular production of reactive oxygen species (ROS), and in particular superoxide [28]. Anti-LOX-1 monoclonal antibody reduces superoxide formation and increases intracellular NO level, suggesting the involvement of LOX-1 in these OxLDL-induced effects [28]. Additionally, OxLDL activates endothelial arginase II (which regulates NO production by competing with eNOS for the common substrate L-arginine) through the dissociation of arginase from the microtubule cytoskeleton, resulting in a decreased NO production and a raise of ROS [29, 30]. The increased arginase activity is inhibited by monoclonal antibody to LOX-1; furthermore, ECs from LOX-1 knockout (KO) mice do not increase arginase activity and exhibit reduced changes in NO and superoxide production in response to OxLDL [30], thus confirming the involvement of LOX-1 in OxLDL-mediated arginase activity. Further, neutralizing antibodies to LOX-1 restore NO-mediated coronary arteriolar dilation in ApoE KO mice [31].

Dysfunctional ECs can also generate vasoconstrictor factors, including endothelin-1 (ET-1) and angiotensin II (Ang II). ET-1 is a potent vasoconstrictor, proinflammatory, and mitogenic peptide produced by injured vascular ECs in response to several stimuli; a tight interaction exists between ET-1 and OxLDL: OxLDL stimulates the generation of ET-1, and ET-1 enhances the uptake of OxLDL in ECs by promoting the expression of LOX-1 [32]. Incubation of ECs with OxLDL increases ET-1 mRNA and protein expression, an effect inhibited by anti-LOX-1 antibody [33].

Renin-angiotensin system may contribute to the endothelial dysfunction; angiotensin-converting enzyme (ACE), which converts Ang I to Ang II, is mainly expressed in ECs [34]. Ang II induces LOX-1 expression and facilitates OxLDL uptake by ECs [35]; in turn, OxLDL increases the expression of ACE in a concentration- and time-dependent manner. The upregulation of ACE expression in response to OxLDL is mediated by LOX-1, as pretreatment of ECs with LOX-1-blocking antibody significantly reduces the OxLDL-induced expression of ACE [36].

### 2.4. LOX-1 Mediates OxLDL-Induced Apoptosis of ECs

Incubation with high concentrations of OxLDL induces cellular changes that may result in cell death. OxLDL may induce both necrosis and apoptosis; the last is a highly regulated process and involves multiple pathways, including ROS generation, caspase and protein kinase activation, alteration of calcium homeostasis, and alteration of proapoptotic/antiapoptotic gene expression [37]. EC apoptosis results in increased vascular permeability to cells and lipids, smooth muscle cell proliferation, and increased coagulation, thus contributing to the development of atherosclerotic lesions.

OxLDL induces EC apoptosis through LOX-1; in fact, inhibition with antisense to LOX-1 mRNA or with chemical inhibitors of LOX-1 significantly reduces the number of apoptotic cells in response to OxLDL [17]. NF-*κ*B activation following EC exposure to OxLDL acts as a signal transduction mechanism in LOX-1-mediated apoptosis, and antisense to LOX-1 significantly inhibits OxLDL-induced NF-*κ*B activation [17].

OxLDL also activates caspase-9 and caspase-3 in ECs and induces the release of mitochondrial activators of caspases, while reducing the expression of the antiapoptotic proteins B-cell lymphoma 2 (Bcl-2) and cellular inhibitor of apoptosis protein 1 (c-IAP-1) [38]. These effects are mediated by LOX-1, as pretreatment of cells with antisense to LOX-1 mRNA significantly decreases OxLDL-induced activation of caspases as well as the percentage of apoptotic cells [38]. These findings indicate that OxLDL through its receptor LOX-1 modulates activity and expression of relevant players in apoptosis.

Fas is a death-receptor that triggers apoptosis when activated by its ligand FasL and is involved in OxLDL-induced apoptosis; in fact, OxLDL sensitizes vascular cells to Fas-mediated apoptosis, upregulating Fas surface expression, while OxLDL-induced apoptosis is reduced by FasL-neutralizing antibodies [37]. LOX-1 activation is involved in these effects, as neutralizing LOX-1 antibody prevents OxLDL-induced activation of Fas-mediated apoptosis and inhibits OxLDL-induced modulation of surface Fas expression in ECs [39].

### 2.5. OxLDL Increases Oxidative Stress through LOX-1 in ECs

High levels of ROS are produced in several disease conditions, including atherosclerosis, and contribute to endothelial dysfunction. ROS are involved in LDL oxidation, and, in turn, OxLDL mediates many of its biological effects by generating more intracellular ROS through the binding to LOX-1; anti-LOX-1 antibody markedly reduces OxLDL-induced ROS formation [23, 40]. OxLDL-induced increase of ROS also results in NF-*κ*B activation [23] and in EC apoptosis [40]; both these effects are mediated by LOX-1, as pretreatment of the cells with LOX-1-blocking antibody significantly reduces ROS production, NF-*κ*B activation, and apoptosis rate in response to OxLDL [23, 40].

Endothelial NADPH oxidase, a multisubunit enzymatic complex, is a major source of ROS in vascular ECs, and OxLDL induces a significant increase of NADPH oxidase-generated ROS in ECs [41]. The binding of OxLDL to LOX-1 activates NADPH oxidase by inducing translocation of specific subunits on the cell membrane, leading to a rapid increase in intracellular ROS such as hydrogen peroxide and superoxide; the latter reacts with intracellular NO, thereby causes intracellular NO level to decrease, and upregulates LOX-1 expression, thus resulting in further increase in ROS production [42]. 

p66^Shc^ is a redox enzyme involved in mitochondrial ROS generation and the translation of oxidative signals into apoptosis; higher levels of p66^Shc^ have been found under pathological conditions and well correlated with oxidative stress in cardiovascular disease [43]. When exposed to OxLDL, ECs increase phosphorylation of p66^Shc^, an effect that is prevented by blockade or molecular silencing of LOX-1 [43], supporting a role for LOX-1 also in this OxLDL-mediated effect. On the other hand, lysophosphatidylcholine (LPC), a relevant component of OxLDL, induced p66^Shc^ activation independently of LOX-1 [43].

In HCAECs, OxLDL promotes intracellular ROS production and induces DNA oxidative damage [44], resulting in the regulation of several transcription factors, including NF-*κ*B and octamer-binding transcription factor-1 (Oct-1). Oct-1 acts as a transcriptional repressor for endothelial enzymes involved in the production of vasoactive molecules, thus providing a link between oxidative DNA damage and impaired function of endothelium upon exposure to OxLDL. Oct-1 is also involved in OxLDL-induced LOX-1 promoter activation and gene expression [45]. Inhibition of LOX-1 attenuates OxLDL-mediated endothelial DNA damage, Oct-1/DNA binding, and reverses impaired production of vasoactive compound [44].

### 2.6. Other Effects of OxLDL Mediated by LOX-1 in ECs

Incubation of ECs with OxLDL modulates the expression of several other genes associated with atherosclerosis, and LOX-1 plays a crucial role in mediating such effects.

Metalloproteinases (MMPs) are a family of matrix degrading enzymes involved in vascular remodeling that contribute to the determination of atherosclerotic plaque stability; their expression and activity are increased in atherosclerotic plaques [46]. OxLDL increases the expression of MMP-1 and MMP-3 mRNA and protein in ECs, without affecting the expression of tissue inhibitor of metalloproteinases (TIMPs) [47], suggesting an OxLDL-induced imbalance between MMPs and TIMPs. LOX-1 activation mediates the modulation of MMPs by OxLDL: LOX-1-blocking antibody prevents the increase of MMPs in response to OxLDL [47]. Similarly, OxLDL enhances MMP-9 production in human aortic ECs, and anti-LOX-1 antibody inhibits this effect [48].

Angiogenesis is a highly regulated physiological process involved in several pathological conditions including inflammation and atherosclerosis and requires disruption of cell-cell contact, EC migration, proliferation, and capillary tube formation. Generation of small amount of ROS, as those induced by low concentrations of OxLDL (<5 *µ*g/mL), seems to be involved in this process. Low concentrations of OxLDL stimulate tube formation from ECs *in vitro. *This effect is inhibited by anti-LOX-1 antibody, that also decreases OxLDL-induced ROS generation, NF-*κ*B activation, and upregulation of vascular endothelial growth factor (VEGF) [49].

The plasminogen activator (PA) is involved in the control of fibrinolysis within the vascular lumen; plasminogen activator inhibitor-1 (PAI-1) attenuates fibrinolysis through inhibition of plasminogen activation and modulates cellular responses; increased expression of PAI-1 has been described in atherosclerosis [50]. OxLDL induces PAI-1 upregulation in cultured ECs through LOX-1, as showen by the inhibitory effect of anti-LOX-1-blocking antibody; this finding suggests an involvement of LOX-1 also in OxLDL-induced thrombotic process [51].

The LDL receptor regulates plasma LDL-cholesterol levels. OxLDL decreases the expression of LDL receptor in a concentration- and time-dependent manner in HCAECs [52]. This effect is mediated by LOX-1, as cell pretreatment with LOX-1-blocking antibody or with antisense to LOX-1 mRNA reduces the effect of OxLDL on LDL-receptor expression [52].

### 2.7. LOX-1 Mediates OxLDL-Induced Effects in Endothelial Progenitor Cells

Endothelial progenitor cells (EPCs) are involved in the regeneration of the injured endothelium and in the neovascularization process, which represents a compensatory mechanism in ischemic disease; atherosclerosis is associated with reduced numbers and impaired functionality of EPCs [53]. OxLDL has a negative effect on EPCs, induces EPC senescence, inhibits VEGF-induced EPC differentiation, decreases EPC number, and impairs their function [54–56]. LOX-1 is expressed in EPCs, and incubation of EPCs with OxLDL increases LOX-1 expression [57], an effect depending on the interaction between OxLDL and LOX-1, as confirmed by the use of an anti-LOX-1 antibody. OxLDL also induces EPC apoptosis in a dose-dependent manner, thus reducing their survival; moreover, OxLDL impairs EPC adhesive, migratory, and tube formation capacities [57]. All these effects are attenuated by pretreatment with a LOX-1 monoclonal antibody. Furthermore, OxLDL reduces eNOS expression and NO production, that may in part explain the inhibitory effect of OxLDL on EPC survival and function [57]. Pretreatment with anti-LOX-1 antibody inhibits all these OxLDL-induced effects.

At low concentrations (<10 *μ*g/mL) OxLDL does not induce apoptosis but accelerates EPC senescence; this effect is significantly attenuated by LOX-1-blocking antibody or by atorvastatin (that reduces LOX-1 expression) [54]. OxLDL significantly reduces telomerase activity (which plays a critical role in cellular senescence) and impairs proliferation and network formation capacity, resulting in cellular dysfunction [54].

## 3. Role of LOX-1 in OxLDL-Induced Smooth Muscle Cell Proliferation and Apoptosis

LOX-1 is expressed also in smooth muscle cells (SMCs) [6]; *in vitro*, several stimuli, including OxLDL and Ang II, can upregulate LOX-1 expression in SMCs [58–60]; *in vivo*, LOX-1 protein is not detectable in the media of noninjured aorta but is present in SMCs two days after vascular injury or after balloon-angioplasty [61]. OxLDL and LOX-1 colocalize with SMCs of human restenotic lesions, suggesting a role for LOX-1 in OxLDL-induced SMC proliferation and restenosis [61].

MicroRNAs are small noncoding RNAs that negatively modulate gene expression through the binding with their mRNA and play a role also in atherosclerosis [60]. MicroRNA let-7g targets LOX-1 gene and inhibits its expression; OxLDL, by inducing the transcription factor Oct-1, reduces let-7g expression. Let-7g mimic reduces OxLDL-induced LOX-1 and Oct-1 upregulation as well as OxLDL-enhanced SMC proliferation and migration [60].

At higher concentrations, OxLDL upregulates LOX-1 expression and induces apoptosis of vascular SMCs [62] (Figure 3), a process that may contribute to atherosclerotic plaque destabilization. OxLDL-induced apoptosis is a consequence of LOX-1 upregulation, as apoptosis is inhibited by a neutralizing anti-LOX-1 antibody. Furthermore, OxLDL increases the expression of the proapoptotic protein Bcl-2-associated X protein (Bax) and inhibits the expression of the antiapoptotic Bcl2. This effect is mediated by LOX-1, as anti-LOX-1 antibody markedly inhibits OxLDL-induced modulation of these two proteins [62]. LOX-1 colocalizes with Bax in human atherosclerotic plaques, particularly in rupture-prone shoulder region, suggesting a role for LOX-1 in the mechanisms that contribute to plaque destabilization [62].

Since SMCs can transform into foam cells, LOX-1 might be a potential player in this process (Figure 3). Treatment of SMCs with LPC increases LOX-1 expression [63], with consequent LOX-1-mediated increase of OxLDL uptake; anti-LOX-1 antibody markedly inhibits the LPC-enhanced OxLDL uptake [63].

Bone marrow cells can potentially originate smooth muscle progenitor cells (SMPCs) that may differentiate into smooth muscle-like cells (SMLCs) in the damaged vessels [64], thus contributing to the atherogenic process. *In vitro*, long-term culture with platelet-derived growth factor PDGF-BB induces the differentiation of SMPCs into SMLCs that express SMC-specific markers [65]; OxLDL inhibits PDGF-BB-induced differentiation of SMLCs, as revealed by the decrease of SMC marker expression in the presence of OxLDL. Furthermore, OxLDL incubation induces lipid droplet accumulation in the cytoplasm and LOX-1 surface expression [65]. Anti-LOX-1 antibody significantly reduces OxLDL uptake by SMLCs, suggesting a role for LOX-1 in the transdifferentiation of SMLCs into foam-like cells [65].

## 4. Role of LOX-1 in OxLDL-Induced Effects in Macrophages

Macrophages internalize OxLDL by several SRs including SR-AI/II, SR-BI, cluster of differentiation 36 (CD36), and LOX-1, resulting in lipid accumulation and transformation into foam cells. LOX-1 is not detectable in freshly isolated human monocytes, but its expression increases in differentiated macrophages [66]. LOX-1 expression in macrophages can be upregulated by several stimuli, including OxLDL, LPC, high-glucose levels, and proinflammatory cytokines [8]. The contribution of LOX-1 in macrophage uptake and degradation of OxLDL under normal conditions is small. No significant differences are observed between wild-type and LOX-1 deficient macrophages [67], probably due to the high expression of other SRs that could mask the contribution of LOX-1. However, in cells stimulated with LPC, LOX-1 expression and OxLDL uptake and degradation increase in wilt type cells but not in LOX-1-deficient cells [67]. These observations suggest that LOX-1 gene inactivation does not markedly modify OxLDL uptake in unstimulated macrophages, as LOX-1 accounts for 5–10% of OxLDL uptake by these cells, but, when LOX-1 is upregulated, internalization of OxLDL increases by more than 40% [67] (Figure 2). Proinflammatory cytokines upregulate LOX-1 and downregulate other SRs (SR-AI/II and CD36), suggesting that, in inflamed microenvironments, where these cytokines are relatively abundant (such as in atherosclerotic lesions), LOX-1 might play a significant role in macrophage OxLDL uptake.

Monocytes may also differentiate into dendritic cells (DCs), a specific type of leukocytes that play a key role in the initiation of innate and adaptive immune responses, and OxLDL affects both DC maturation and migration [68, 69]. LOX-1 is highly expressed on mature DCs and significantly contributes to OxLDL uptake, as anti-LOX-1 antibody reduced OxLDL uptake by 48% [70].

## 5. Role of LOX-1 in Platelet Activation

Platelets express several SRs, some of which are constitutively expressed (including CD36), while LOX-1 appears on the surface of platelets on activation [71]; thus, in resting platelets, CD36 mediates OxLDL binding to platelets, while, in activated platelets, in which OxLDL binding is increased compared to resting cells, binding of OxLDL is mediated by both CD36 and LOX-1 [71]. Activated platelets internalize significant amount of OxLDL compared with resting platelets [72] (Figure 3); OxLDL induces platelet activation, increases their ability to adhere to ECs, induces an inflammatory response, and leads to platelet accumulation after vascular injury. In fact, OxLDL-positive platelets induce adhesion molecule expression on ECs, reduce regeneration of ECs, and induce foam cell formation [72], suggesting that OxLDL-activated platelets may contribute to vascular inflammation by several mechanisms.

Dysfunctional endothelium exhibits procoagulant and adhesive properties to platelets, and endothelial LOX-1 plays a major role in the platelet-endothelium interaction thus enhancing endothelial dysfunction. In fact LOX-1 binds also anionic phospholipids, including those present on the surface of apoptotic cells or activated platelets [73], thus working as an adhesion molecule for platelets. OxLDL partially inhibits the binding of platelets to LOX-1, indicating a high affinity of platelets for this receptor [73]. The binding of activated platelets to endothelial LOX-1 induces the release of ET-1, further supporting the induction of endothelial dysfunction [73]. Furthermore, the binding of activated platelet to endothelial LOX-1 induces ROS generation followed by a reduction of NO bioavailability; all effects are prevented by anti-LOX-1 antibody [74].

## 6. LOX-1 and Atherosclerosis: Experimental Evidences

As previously described, LOX-1 mediates many of the effects of OxLDL, that is, EC growth, dysfunction, adhesion, and activation of monocytes/macrophages, and all critical features of atherosclerosis. LOX-1 mRNA has also been found in human atherosclerotic plaques, while negligible amounts are detectable in unaffected aortas [11]. The key relevance of LOX-1 in atherosclerosis has been demonstrated by key experiments in LOX-1 KO mice crossed to a mouse model prone to develop atherosclerosis such as the LDL-R KO mouse. Feeding these mice with a cholesterol-rich diet results in a reduced binding of OxLDL to the aortic endothelium and a preservation of endothelium-dependent vasorelaxation after treatment with OxLDL compared to wild type animals [12]. More importantly, LOX-1 KO/LDL-R KO animals show a significant reduction in atherosclerosis compared with LDL-R KO mice [12]. This is associated with reduced NF-*κ*B expression as well as decreased inflammatory markers and also with an increased eNOS expression, suggesting an improved endothelial function [12]. Conversely, mice overexpressing LOX-1 on ApoE KO background show a dramatic increase in atherosclerosis compared to the nontransgenic mice [13]. In hypercholesterolemic rabbits, LOX-1 expression is detected mainly in atherosclerotic plaques with a thinner fibromuscular cap and is localized to the lipid core where it correlates with tissue factor expression and apoptosis [75], potentially connecting LOX-1 with plaque destabilization and rupture. In line with this, LOX-1 expression is increased in unstable plaques when the AMI-prone Watanabe heritable hyperlipidemic rabbit and control rabbits are injected with an antibody tracing LOX-1 [76].

Among the critical player in atherogenesis, the activation of the rennin-angiotensin system and the consequent generation of Ang II are thought to be critical factors. Ang II via its type 1 receptor (AT1 receptor) also upregulates the expression of LOX-1 mRNA, and OxLDL via LOX-1 upregulates the expression of AT1 receptor [77]. Of note hypertensive rats present an upregulation of LOX-1 expression mainly in vascular ECs [78–80]. The mutually facilitatory cross-talk between LOX-1 and AT1 receptors may explain the coexistence of multiple risk factors in the same patient and the increase in atherosclerosis risk with the presence of multiple risk factors. In agreement with this assumption, LOX-1 expression is increased in several animal models of cardiometabolic disorders, including streptozotocin-induced diabetes [81] or ischemia-reperfusion (I/R) injury [82]. LOX-1 deficiency is associated with a more preserved left ventricular function following I/R injury which resulted in a reduced oxidative stress, collagen deposition, and fibronectin expression thus resulting in a significant decrease in myocardial injury as well as in the accumulation of inflammatory cells [83, 84].

Altogether, the results from experimental atherosclerosis support a proatherogenic role for LOX-1.

## 7. Genetics of LOX-1 and Atherosclerosis

LOX-1 is encoded by the oxidized low-density lipoprotein (lectin-like) receptor 1 (OLR1) gene, mapped to chromosome 12p13 [85]. The association of polymorphisms in the human OLR1 gene with the susceptibility to myocardial infarction (MI) has been reported [85, 86]. In particular, six single nucleotide polymorphisms (SNPs), located within introns 4, 5, and 3′ UTR (untranslated region), that are comprised in a linkage disequilibrium (LD) block are strongly associated with the elevated risk to develop MI [86]. The observation that the SNPs related to an increased risk of MI do not affect the coding sequence of the gene suggests the possibility that the SNPs could give rise to a functional product such as messenger RNA (mRNA) isoforms as a consequence of alternative splicing. Indeed it has been shown that the SNPs located in the LD block regulate the level of the new fully functional transcript by modulating the retention of exon 5 of the OLR1 gene [85]. The alternative splicing of OLR1 mRNA leads to different ratios of LOX-1 full receptor and LOXIN (lack of exon 5), an isoform lacking part of the C-terminus lectin-like domain which is a functional domain. LOXIN, through heterooligomerization with LOX-1 [87], blocks the negative effects of LOX-1 activation, and this variant has a functional role on plaque instability and therefore in the pathogenesis of MI [86]. *Ex vivo* data show that macrophages from subjects carrying the “nonrisk” allele at OLR1 gene display increased expression of LOXIN resulting in protection against OxLDL-mediated apoptosis [87].

Later, an SNP on exon 4, rs11053646 (G501C), which leads to an amino acidic substitution (lysine to asparagine at position 167, K167N) has been studied. Of note, basic residues in the lectin domain are important for strengthening the ligand binding; substitution of this residue (K167N) causes a change on the positive isopotential surface and thereby results in reduced binding and internalization of OxLDL [88]. This study, performed on CV-1 (simian) in Origin and carrying the SV40 (COS-1) cells overexpressing either GG (KK) or CC (NN) LOX-1, showed that GG (KK) COS-1 cells bind and internalize less OxLDL than CC (NN) [88].

The K167N SNP has been identified among others in the *ORL-1 *gene to be associated with acute MI and coronary artery disease (CAD) [89–91]. The frequencies of the KK genotype and the K allele are higher in the CAD group than in controls (*P* < 0.05), while the opposite is true for NN genotype (*P* < 0.05) [92]. The relevance of LOX-1 SNPs has been tested also in relation to markers of atherosclerosis. Among them, ultrasound detection and quantification of the common carotid artery wall thickness (intima-media thickness, IMT) are considered a surrogate marker of subclinical atherosclerosis [93, 94]. The association between the nonsynonymous substitution K167N (rs11053646) and IMT has been tested in 2,141 samples from the Progression of Lesions in the Intima of the Carotid (PLIC) study (a prospective population-based study) [95]. Significantly increased IMT has been observed in male carriers of the minor C (N) allele compared to GC and GG (KN and KK) genotypes [95]. A gender-specific association has been also described between the C (N) allele and prevalence of carotid plaque also in a cohort of Dominican-Hispanic origin [96].

Functional analysis on macrophages suggests a decreased association to OxLDL in NN carriers compared to KN and KK carriers which is also associated with a reduced OLR1 mRNA expression [95]. Macrophages from NN carriers present also a specific inflammatory gene expression pattern compared to cells from KN and KK carriers [95]. How these data, obtained with human primary macrophages, relate with those obtained in a transfected fibroblast-like cell line derived from monkey kidney tissue is unclear, and further studies are warranted to clarify this issue [88].

In summary, genetic alterations favoring LOXIN isoform production coupled to the observation that this isoform exerts a dominant-negative effect on LOX-1 function make it an attractive new target for prevention and treatment of initiation, progression, and clinical consequences of atherosclerosis such as plaque instability, acute myocardial infarction, and ischemia reperfusion injury.

## 8. Concluding Remarks

OxLDL plays multiple roles in atherosclerosis; LOX-1 scavenger receptor mediates many of the OxLDL-induced effects, and the OxLDL-LOX-1 interaction can alter the expression of several genes, resulting in the induction of cellular dysfunction, proliferation, and apoptosis. A recent study showed that LOX-1 inhibition in ApoE KO mice using a schizophyllan-based antisense oligonucleotide therapy resulted in reduced LOX-1 expression in the arterial wall [97]. Blocking the expression and/or function of LOX-1 results in the improvement of cellular functions and reduction of atherosclerotic lesion formation, suggesting that LOX-1 might be an attractive therapeutic target for the management and the prevention of atherosclerosis.

## Figures and Tables

**Table 1:** LOX-1 inducers.

Proinflammatory cytokines	
Tumor necrosis factor *α* (TNF*α*)	
Interleukin-1 (IL-1)	
Interferon *γ* (IFN*γ*)	
Lipopolysaccharide (LPS)	
C-reactive protein (CRP)	
Modified lipoproteins	
OxLDL (copper-oxidized LDL)	
15-Lipoxygenase-modified LDL	
15-Lipoxygenase-modified HDL_3_	
Glycoxidized-LDL	
Lysophosphatidylcholine (LPC)	
Palmitic acid	
Hypertension-related stimuli	
Angiotensin II	
Endothelin-1	
Fluid shear stress	
Hyperglycemic stimuli	
High glucose	
Advanced glycation end-products (AGEs)	
Other stimuli	
Homocysteine	
Free radicals	

**Table 2:** LOX-1 ligands.

Modified lipoproteins	Other ligands
OxLDL (copper-oxidized LDL)	Apoptotic cells
15-Lipoxygenase-oxidized LDL	Activated platelets
15-Lipoxygenase-oxidized HDL_3_	Advanced glycation end-products (AGEs)
Glycoxidized LDL	
Delipidated OxLDL	
HOCl-modified HDL	
